# Influence of loss- and restoration-oriented stressors on grief in times of COVID-19

**DOI:** 10.1038/s41598-023-46403-6

**Published:** 2023-11-09

**Authors:** Svenja Palm, Bettina K. Doering, Thomas Kubiak, Katharina Geschke, Andreas Fellgiebel, Alexandra Wuttke

**Affiliations:** 1 Center for Mental Health in Old Age (ZpGA), Landeskrankenhaus (AöR), Hartmühlenweg 2-4, 55122 Mainz, Germany; 2 Institute of Psychology, Kiel University, Grippstraße 2, 24118 Kiel, Germany; 3 Institute of Psychology, Johannes Gutenberg University, Binger Str. 14-16, 55122 Mainz, Germany; 4 Department of Psychiatry and Psychotherapy, University Medical Center Mainz, Untere Zahlbacher Straße 8, 55131 Mainz, Germany; 5 Hospital for Psychiatry, Psychosomatic and Psychotherapy, Agaplesion Elisabethenstift, Landgraf-Georg-Str. 100, 64287 Darmstadt, Germany; 6 Center for Mental Health, University Hospital Würzburg, Margarete-Höppel Platz 1, 97080 Würzburg, Germany

**Keywords:** Public health, Psychology

## Abstract

This study aimed to examine the influence of COVID-specific stressors on cross-sectional and longitudinal bereavement outcomes. According to the Dual Process Model of grief these stress-inducing factors can relate to the loss (loss-oriented stressors) or to manage everyday life (restoration-oriented stressors) and require coping in the grief process. A total of 491 participants (94.1% female; 43.92 years on average; 44.4% loss of a parent) were included at the first measurement time point (T0), of whom 99 individuals also participated in a follow-up assessment 6 months later (T1). Participants frequently reported loss-oriented (on average 7.30 out of 21 queried) and restoration-oriented stressors (on average 6.99 out of 19 queried). Cross-sectionally, higher acute grief intensity was associated with a higher number of loss-oriented stressors, poorer mental well-being, and sociodemographic variables. This effect disappeared longitudinally, with only acute grief intensity and poorer mental well-being at T0 predicting higher prolonged grief at T1. Common resilience factors did not buffer the effects of the pandemic on grief. Loss-oriented stressors seem to be especially relevant for understanding grief and might be a mediator of higher long-term grief. The findings suggest that COVID-specific strains need to be specifically taken into account in the support of bereaved individuals.

## Introduction

As most people experience the loss of a significant other during their life, grief can be considered a normal and natural human experience^1^. For many bereaved people, grief means intense suffering, but despite this painful experience, the majority show resilience in their grief and return to their previous level of psychosocial functioning. Only a minority of individuals suffer from persistent grief-related mental health impairments^2^.

One of the most widely used psychological models of grief is the Dual Process Model of coping with bereavement^3^ (DPM), which explains the grieving process as a long-term coping process with individual stressors. Stressors are understood here as events that trigger unpleasant feelings in the bereaved person and must somehow be coped with. These stressors can consequently vary greatly from one individual to another, but can be classified into two broad categories: Stroebe and Schut^4^ define loss-oriented stressors as stressful aspects of the loss experience itself (e.g., visiting the grave of the deceased person). Restoration orientation, on the other hand, refers to stressors that arise in the attempt to find one's way back to everyday life following a loss (e.g., taking over tasks of the deceased in everyday life). Adaptation to bereavement involves an oscillation between these processes over a longer period of time. Oscillation is described here as a process in which individuals turn to or avoid loss- or recovery-oriented aspects at times, and in which there may also be times when grief does not play a major role for affected individuals^4^. Stroebe and Schut^5^ assume that negative mental and physical health consequences result from an overload of stressors, which can occur through an individually overwhelming exposure to loss-oriented, restoration-oriented, or everyday stressors.

COVID-19, as a global pandemic, has been associated with various experiences of loss at multiple levels and may have impeded the ability to cope with stressful situations^6^. At the outset of the pandemic, various research groups warned that the burden of grief could become heavier for individuals and for health systems. For instance, the pandemic-related restrictions may have increased both loss-oriented stressors (e.g., extreme end-of-life stress, unexpected deaths) and restoration-oriented stressors (e.g., financial worries, fear of infection)^7^. Moreover, during the pandemic, bereaved individuals might have been faced with a loss of potential resources to strengthen resilience (daily structure, positive activities), a lack of social support, and a decrease in their locus of control. It was also hypothesized that experiences of disenfranchised grief^8^, i.e., the experience of grieving losses that are not acknowledged or supported by social systems^9^, could become more prevalent. Furthermore, a concomitant drastic increase in the number of bereaved persons due to the assumed strong increase in mortality was predicted—which, combined with the difficulties in coping with grief, was expected to lead to widespread burdens in society^8^.

A detailed outline of the hypothesized COVID-specific stress factors was compiled by Stroebe and Schut^7^ in a review of the literature on adaptation to bereavement during the pandemic, by applying the perspective of recovery- and loss-oriented stressors from the DPM. However, at the onset of the pandemic, these COVID-specific factors were theoretically derived, i.e., from studies of previous pandemics and considerations of possible consequences of measures to mitigate the pandemic impact. Subsequent studies have meanwhile gathered first evidence to support the hypotheses on the impact of measures to curb the pandemic as well as general pandemic-related stressors: By examining qualitative responses from two national surveys of bereavement during the pandemic Torrens-Burton et al.^10^ supported the utility of the DPM as a conceptual model to understand the impact of the pandemic on grief. Many of the theoretically assumed stressors (e.g., “no goodbyes possible”) and assumed emotional reactions (e.g., guilt, shame) occurred in the respondents' report. Research conducted with counselors and psychotherapists further indicates that the assumed issues like lack of social support or the omission of traditional bereavement rituals played a prominent role in the counseling and therapy of bereaved individuals during the COVID-19 pandemic^11,12^. This also supports the idea that the changes brought about by the pandemic exerted unique stress on the bereaved as well. Supporting findings are meanwhile also found for the assumption that the COVID-19 circumstances might exacerbate grief: Selman et al.^13^ found evidence that pandemic-related stressors (death from SARS-CoV-2, lack of support from professionals, social isolation, reduced social contacts) could be associated with a poorer end-of-life experience. Recently, Van Schaik et al.^14^ conducted a review of the available literature regarding the effect of the pandemic on grief experiences; their overview likewise found evidence to support the hypothesis that the COVID-19 pandemic has had a significant impact on mourning processes: Findings on the emotional and stressful response to losses during the COVID-19 pandemic, losses to SARS-CoV-2 infection, changes in social support, and mourning rituals were described in detail. At the same time, Van Schaik et al.^14^ pointed out that the findings suggest that mourners continue to oscillate between loss- and restoration-oriented attitudes during this time, and that positive aspects of mourning during the COVID-19 pandemic can also be found.

Another assumption at the outset of the pandemic was that the containment measures would also result in the loss of important resources for coping with grief, such as social support. Only recently, Bonanno^15^ described an interesting paradox: although the majority of bereaved individuals manage to cope well with grief, and various predictors have now been associated with resilient outcomes, the prediction of resilient trajectories remains poor. In most cases, results indicate only moderate effects or mixed findings on individual protective factors. For this reason, the author assumes a flexible adaptation process in which coping mechanisms can change over time. With regard to the oscillation process of the DPM for coping with grief, this would be an interesting explanation for resilient grief trajectories. As a result, testing the hypothesis regarding the loss of important resource appears to be rather difficult. Nevertheless, it would be interesting to see to what extent resources such as social support, resilience or self-efficacy would be impaired in times of uncertainty.

Considering that a widespread burden of grief was anticipated at the outset of the pandemic, determining the proportion of those affected, who actually experienced such stressors, would be important for further investigations. To the best of our knowledge, no study so far has quantitatively investigated the hypothesized COVID-specific loss-oriented and restoration-oriented stressors of the DPM in a general sample of bereaved individuals and examined their influence on grief intensity. Acknowledging the burdens of bereavement during the pandemic would also be important socially, to provide space for bereaved individuals to talk about experiences and to provide the social support that may have been denied during the pandemic. Moreover, the majority of the reported findings are based on cross-sectional studies. The assumed dynamic of grief coping requires a longitudinal research design to be able to assess the impact of the stressors on grief. It also remains unclear to what extent important resources such as resilience, social support, or self-efficacy have been diminished by pandemic containment measures and whether this might have an impact on grief. We therefore set out to examine the influence of COVID-specific loss- and restoration-oriented stressors, alongside general measures of mental health (mental well-being and perceived stress) as well as protective factors (resilience, social support, and self-efficacy), on grief from a cross-sectional (research question 1) and longitudinal perspective (research question 2).

## Methods

### Study procedure

**Figure 1:**
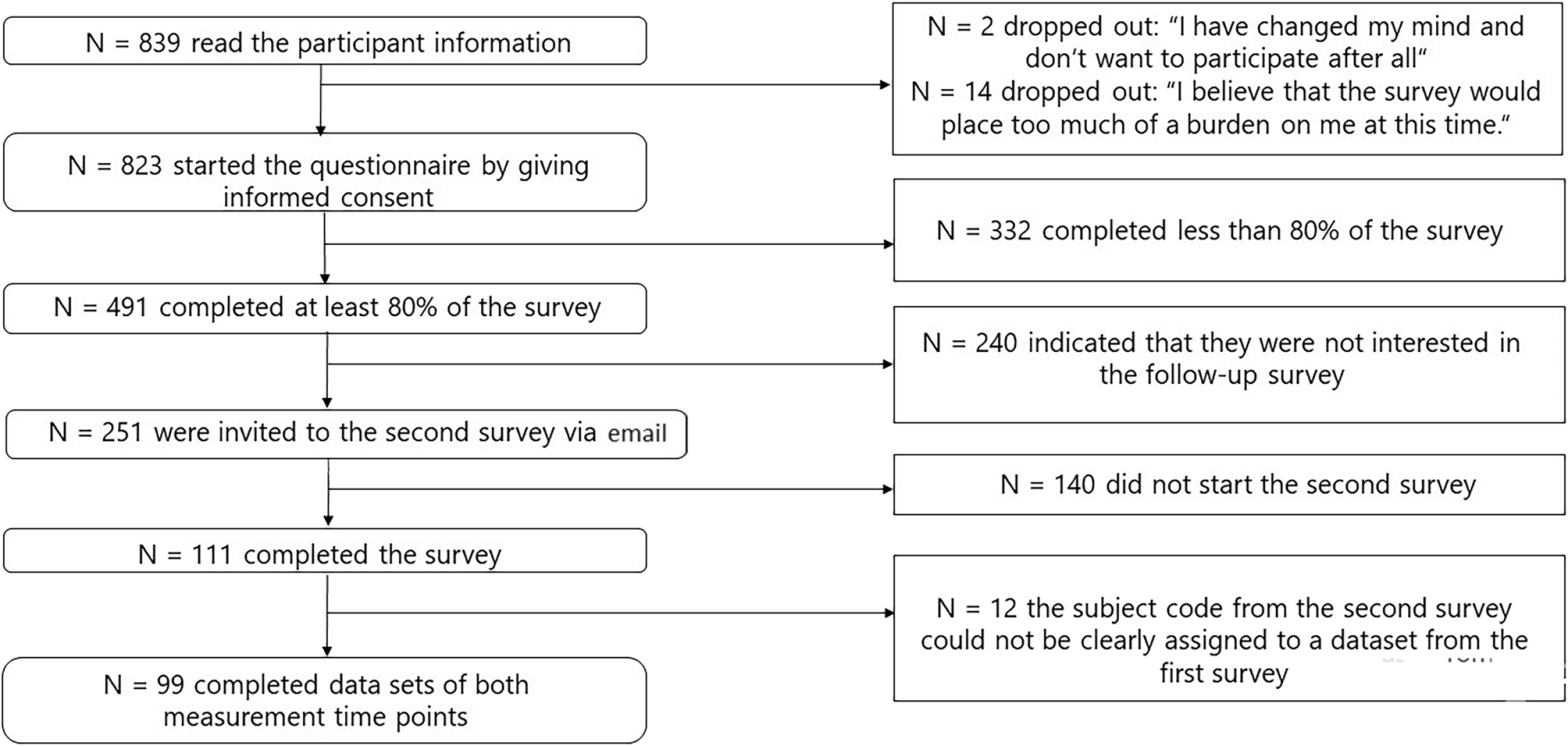
Flowchart of participation. The development of the number of participants with the respective reasons for dropouts is illustrated.

The survey consisted of two measurement time points conducted online via the SoSci survey platform (T0: July 1–December 15, 2021; T1: January 15–July 15, 2022). Participants were invited via email to take part in the second survey after approximately 6 months. Inclusion criteria were age ≥ 18 years and having lost a significant other during the COVID-19 pandemic (from March 2020 onwards). We recruited participants through posts in online bereavement groups and using Google ads with specific search terms. Additionally, we contacted bereavement groups and hospices throughout Germany with the request to share the study information. Study participation was voluntary and no financial compensation was provided. We followed the recommendations of Smith et al.^16^ for the responsible and ethical conduct of online research in bereaved samples: Immediately after providing informed consent, participants received a self-care cue, and at the latest 1 week after completing the survey, they received a “check-in” email thanking them for their participation, validating uncomfortable feelings, and offering to arrange a telephone counselling call with a psychotherapist from the team. Two of the participants accepted this offer at T0. To gain better insight into reasons for not participating, we included two additional items (see Fig. 1). The study was approved by the ethics committee of the State Chamber of Physicians of Rhineland-Palatinate (Landesärztekammer RLP, reference number: 2021-15784_1) and preregistered at the German Clinical Trials Register prior to implementation (DRKS; registration number DRKS00024908). All participants provided informed consent prior to study participation. Our research has been performed in accordance with the Declaration of Helsinki.

### Study sample

A total of 823 persons started the online survey. Of these, 332 participants completed less than 80% of the survey and were thus excluded. This resulted in a final sample of 491 participants at the first measurement time point (T0). Of these, 361 people (73.52% of T0 participants) expressed interest to participate in the second assessment 6 months later (T1) by clicking on an item in the survey. To prevent linking the survey data with personal data, the email address for the T1 survey was collected via a separate query. 251 individuals (51.12% of T0 participants) provided their email address at T0. A total of 99 participants (20.16% of T0 participants) were included in the T1 survey (see Fig. 1 and Table 2 for detailed participation information). As sociodemographic data we assessed gender, age, education, occupation status, relationship status, housing situation as well as the relationship with the deceased.

### Measurements

Based on considerations by Stroebe and Schut^7^ on the specific loss- and restoration-oriented stressors during the COVID-19 pandemic, we created an item pool culturally adapted to Germany (e.g., taking into consideration the general health insurance system in Germany, which protects against impoverishment due to medical treatment), which is presented in Table 1. The items assessed a total of 40 stressors (21 loss-oriented stressors, 19 restoration-oriented stressors), which were dichotomized (“agree”/“disagree”). We then calculated the frequency of their occurrence as well as the individual sum value of endorsed stressors. The main outcome, grief intensity, was assessed using two different questionnaires. The German version of the Texas Revised Inventory of Grief (TRIG)^17^ was used at T0 to assess the extent and intensity of acute grief reactions. The TRIG is an established questionnaire for measuring grief and has shown good psychometric properties^18,19^. To assess the presence of prolonged grief symptoms, we used the German version of the International Prolonged Grief Disorder Scale (IPGDS)^20^. The IPGDS is a questionnaire designed specifically to test for the criteria of prolonged grief disorder newly introduced in the ICD-11 and has likewise shown good psychometric properties^21^. If respondents already reported at the first measurement point that the loss occurred more than 6 months ago, the questionnaire was administered at both measurement points. In the case of losses that occurred more recently, respondents completed the IPGDS only at T1. Due to the widely discussed burdens of the COVID-19 pandemic, two additional questionnaires related to mental health and stress were also presented: We included the 5-item World Health Organization Well-Being Index (WHO-5)^22^ and the German version of the Perceived Stress Scale (PSS)^23^ to provide an overall estimate of perceived stress levels. To assess possible protective factors, we additionally included the Brief Resilience Scale (BRS)^24^ and the German Brief Perceived Social Support Questionnaire (F-SozU)^25^. As it was suspected that the grieving process during the COVID-19 pandemic was associated with loss of control and loss of routines^7^, we added self-efficacy as an exploratory protective factor in the pandemic measured by the short form of the German-language General Self-Efficacy Short Scale (ASKU)^26^. All of these questionnaires have been validated and have acceptable psychometric properties^27–31^. At the second measurement (T1), only an abbreviated questionnaire battery consisting of the IPGDS, PSS, WHO-5, and BRS was assessed. The control variables of interest were each addressed using a single question (relationship quality at T0: How would you rate the quality of your relationship with the deceased person? (range: 0 (very poor)–100 (very good)), and at T1: Has another significant other of yours died since the last survey? yes/no).

**Table 1:** Item pool of COVID specific stressors adapted from the Dual Process Model and number and percentage of participants endorsing each item.

Loss-oriented stressors	N	%	Restoration-oriented stressors	N	%
L01: My significant other died of COVID-19 even though s/he was not in an at-risk group	28	5.7	R01: I got into financial difficulties due to the COVID pandemic	87	17.8
L02: My significant other had to suffer before death due to COVID restrictions	175	35.7	R02: I perceived the separation from my social environment as stressful	393	80.2
L03: The medical staff did not provide me with sufficient information about my relative's health condition	181	36.9	R03: I feel like I am being judged by others because my significant other passed away from/with COVID	17	3.5
L04: The deceased spent the time before death in isolation due to COVID measures	213	43.5	R04: I have had to quarantine in the past due to contact with people with COVID	107	21.8
L05: I perceived the circumstances of the death due to COVID measures as inhumane	145	29.6	R05: I couldn't really maintain social contacts through other mediums like phone calls or video chats	123	25.1
L06: I perceived the circumstances of the death as traumatic due to COVID measures	165	33.7	R06: Because of the COVID pandemic, there was more conflict and tension in my family	174	35.5
L07: During the COVID pandemic, several of my significant other passed away	205	41.8	R07: I felt restricted in my leisure time by the COVID measures	359	73.3
L08: The media coverage of COVID constantly reminds me of my loss	164	33.5	R08: The future worries me a lot	317	64.7
L09: Physical contact with the deceased prior to death was prohibited due to COVID restrictions	247	50.4	R09: Because of the pandemic, there was more conflict and tension among my friends	152	31.0
L10: I feel that my relative was not well cared for in the institution due to the situation during COVID	96	19.6	R10: I could not integrate positive activities into my daily schedule during lockdown	261	53.3
L11: I was unable to visit my significant other before death due to access or travel restrictions	168	34.3	R11: When my relative was sick with COVID, I was very afraid of infecting others	59	12.0
L12: I was unable to attend the funeral due to COVID	24	4.9	R12: I have tested positive for COVID in the past	46	9.4
L13: My significant other died in intensive care	153	31.2	R13: I did not do any physical activity during the lockdown	240	49.0
L14: My significant other passed away alone	162	33.1	R14: I was very afraid of catching COVID	202	41.2
L15: The death caught me unawares	145	29.6	R15: I long for the "normalcy" of my life before the pandemic	390	79.6
L16: I was not informed of the death in a timely manner	77	15.7	R16: I lost my job during COVID	32	6.5
L17: I feel I did not spend enough time with the now deceased person because of the pandemic	126	25.7	R17: I perceived the COVID-related changes at work negatively	318	64.9
L18: I could not say goodbye to the person	262	53.5	R18: I had to reduce my working hours	66	13.5
L19: Due to the COVID pandemic, I am not able to receive the same support from my environment as would otherwise be possible	231	47.1	R19: I had difficulties due to lack of childcare	59	12.0
L20: I avoid dealing with loss when possible	159	32.4			
L21: I perceived the circumstances of the funeral as burdensome	307	62.7			

### Statistical analyses

Data analysis was performed using SPSS (Version 23 V5 R). Participants’ total scores on the respective questionnaires were calculated. To examine the comparability between participants who only completed T0 and those who completed both measurements with regard to age, sex, time since loss, age of deceased, and questionnaire scores, we conducted a one-factor ANOVA. The normal distribution of the scores for both outcomes (TRIG at T0 and IPGDS at T1) was tested using the Shapiro Wilk test. As the TRIG showed a significant non-normal distribution, the subsequent regression models were computed using bootstrapping with 10,000 samples (as recommended by Hesterberg^32^). For the main analysis, multiple multivariable linear regressions were calculated to predict the two outcomes (acute grief (TRIG; T0), prolonged grief (IPGDS; T1)). Predictors were added stepwise.

In the cross-sectional analyses, in a first step, we tested the predictive value of sociodemographic and loss-related information (age, age of deceased, time since loss, relationship quality, natural cause of death, death due to SARS-CoV-2 infection). COVID-specific stressors were added to the model in the second step (loss-oriented stressors, restoration-oriented stressors). In the next model, we additionally tested mental well-being and perceived stress (WHO-5 at T0, PSS at T0), and in the final model we included protective factors (BRS at T0, ASKU at T0, FSozU at T0). In the longitudinal analysis, in addition to the sociodemographic and loss-oriented information, in the first step of the analysis we also included whether another close person had died since the last survey. Acute grief intensity at T0 (TRIG at T0) was additionally included in the second step of the longitudinal analysis, as the acute grief reaction is known to be a relevant predictor of later grief^33^. In the third step, COVID-specific stressors were added, and the fourth step additionally included mental well-being and perceived stress. Protective factors (BRS at T0, ASKU at T0, FSozU at T0) were included in the fifth step in the final longitudinal model. The calculation of regression models was terminated if the inclusion of further predictors did not result in any further significant improvement of the model. For all analyses, the significance level was set at α = 0.05. To enhance the robustness of the longitudinal analyses, we tested the above described T1 analyses on a dataset with imputed data. For this purpose, we used the data of all individuals who had expressed interest in a follow-up survey at T0 (n = 361). For those of them who had participated only at T0, multiple imputations were used to estimate the value of prolonged grief intensity (IPGDS) and a recent death (yes/no) at T1 from variables which were not used for the main analysis (gender, school degree, occupation, income, relationship status, number of own children, housing situation, residential area, religiousness, relationship with the deceased, cause of death). A set of five imputations were performed, for which a pooled value of the variables of interest (mean of IPGDS, mode of “recent death”) was calculated. The stepwise regressions described previously were then performed. The comprehensive results of these analyses can be found in the supplementary material.

## Results

### Sociodemographic and loss-related characteristics

The majority of participants were female (n = 462, 94.1%) and the mean age was 43.92 years (SD: 11.77, range: 18–80). In terms of educational level, about one third (n = 180, 36.7%) had achieved a medium-track school-leaving qualification as their highest educational attainment. Another 21.4% (n = 105) had a university degree, and the third most commonly reported by respondents was a lower-track school-leaving qualification (n = 78, 16.0%). The majority of respondents were employed, with 169 (34.42%) working part-time and 213 (43.4%) working full-time. More than half of the respondents (n = 286, 58.2%) lived with their family and 43.2% were married. The participants had most often lost a parent (n = 218, 44.4%), followed by a spouse (n = 72, 14.7%). The deceased were on average 67.67 years old at the time of death (SD = 18.25, Min = 0, Max = 101). The most prevalent cause of death was “natural cause” (n = 263, 53.6%); 98 participants (20.0%) lost a significant other to infection with SARS-CoV-2. At T0, the loss had occurred on average 265.30 days ago (SD = 144.97, Min = 1, Max = 609).

When comparing the sample that only participated at T0 with participants who also took part in the longitudinal assessment, those who also participated in the T1 survey were on average older (M = 46.18, SD = 12.12; F(1, 488) = 4.61, p = 0.032). Table 2 presents an overview of all sociodemographic data separately for the two subsamples (participation at T0 only, participation at T0 and T1). Descriptively, individuals who also participated at follow-up were more likely to have a university degree and less likely to have lower-track school-leaving qualifications as their highest educational attainment. Moreover, they were less likely to be currently unemployed or not working but more likely to be widowed and living alone, and showed a higher probability of loss due to SARS-CoV-2 infection. Further information about the psychometric values in the two subsamples is provided in Table 3.

**Table 2:** Overview of sociodemographic data in the total sample as well as separated by the participants of T0 only and participants of both measurement times.

Variable	Characteristics	N (%)_total_	N (%)_T0 only_	N (%)_T0 and T1_
Gender	Female	462 (94.1)	369 (94.1)	93 (93.9)
	Male	29 (5.9)	23 (5.9)	6 (6.1)
Education	No school-leaving qualification	4 (0.8)	4 (1.0)	0 (0.0)
	Lower-track school	78 (16.0)	70 (17.9)	8 (8.1)
	Medium-track secondary school	180 (36.8)	147 (37.7)	33 (33.3)
	Vocational School	44 (9.0)	37 (9.5)	7 (7.1)
	Higher-track secondary school	73 (14.9)	56 (14.4)	17 (17.2)
	University degree	105 (21.4)	71 (18.2)	34 (34.3)
	PhD	5 (1.0)	5 (1.3)	0 (0.0)
	Not answered	2 (0.0)	2 (0.1)	0 (0.0)
Occupation	Working part time	169 (34.4)	133 (34.0)	36 (36.4)
	Working full time	213 (43.4)	165 (42.2)	48 (48.5)
	Unemployed	19 (3.9)	18 (4.6)	1 (1.0)
	Not working	46 (9.4)	41 (10.5)	5 (5.1)
	Retired	43 (8.8)	34 (8.7)	9 (9.1)
	Not answered	1 (0.0)	1 (0.0)	0 (0.0)
Relationship status	Single	89 (18.1)	70 (17.9)	19 (19.2)
	In a steady partnership	83 (16.9)	70 (17.9)	13 (13.1)
	Married	212 (43.2)	169 (43.1)	43 (43.4)
	Divorced	27 (5.5)	23 (5.9)	4 (4.0)
	Widowed	80 (16.3)	60 (15.3)	20 (20.2)
Housing situation	Living alone	128 (26.1)	96 (24.5)	32 (32.3)
	Living with family	286 (58.2)	233 (59.4)	53 (53.5)
	Living in a shared apartment	15 (3.1)	12 (3.1)	3 (3.0)
	Other	62 (12.6)	51 (13.0)	11 (11.1)
Relationship with the deceased	Grandparent	63 (12.8)	51 (13.0)	12 (12.1)
	Parent	218 (44.4)	171 (43.6)	47 (47.5)
	Sibling	19 (3.9)	15 (3.8)	4 (4.0)
	Own child	24 (4.9)	20 (5.1)	4 (4.0)
	Spouse	72 (14.7)	55 (14.0)	17 (17.2)
	Friend	15 (3.1)	11 (2.8)	4 (4.0)
	Parent-in-law	24 (4.9)	23 (5.9)	1 (1.0)
	Someone else	56 (11.4)	46 (11.7)	10 (10.1)
Cause of death	Natural cause of death	263 (53.6)	211 (53.8)	52 (52.2)
	SARS-CoV-2 infection	98 (20.0)	73 (20.3)	25 (25.3)
	Accident	14 (2.9)	12 (3.1)	2 (2.0)
	Violent death	17 (3.5)	12 (3.1)	5 (5.1)
	Other cause of death	99 (20.2)	84 (21.4)	15 (15.2)

**Table 3:** Comparison of the two subsamples (participation at T0, participation at both measurement times) regarding psychometric assessments.

Variable	Participants of T0 only				Participants of T0 and T1				Test Statistics	
	Mean	SD	Min	Max	Mean	SD	Min	Max	F (df)	p
TRIG (T0)	2.36	0.78	1.00	4.88	2.47	0.76	1.00	4.44	1.70 (1, 154.15)	0.194
IPGDS (T1)					36.27	11.20	13.00	63.00		
WHO-5	7.49	5.28	0.00	25.00	9.08	5.98	0.00	22.00	5.82 (1, 141.73)	0.017
PSS	23.48	6.71	1.00	40.00	21.08	8.00	2.00	39.00	7.52 (1, 136.66)	0.007
BRS	2.71	0.82	1.00	5.00	2.95	0.93	1.00	5.00	5.47 (1, 142.38)	0.021
FSozU	22.28	5.53	6.00	30.00	23.66	5.25	9.00	30.00	5.25 (1, 162.28)	0.023
ASKU	3.65	0.79	1.00	5.00	3.83	0.76	1.33	5.00	4.13 (1, 159.51)	0.044
Loss-oriented stressors	7.40	3.19	1.00	16.00	6.89	3.08	1.00	16.00	2.15 (1, 155.28)	0.145
Restoration-oriented stressors	7.09	2.50	0.00	14.00	6.58	2.65	1.00	15.00	3.066 (1, 145.15)	0.082

Note that for all questionnaires, higher scores indicate higher trait manifestation, with the exception of the TRIG, where lower scores indicate higher grief intensity. *TRIG* Mean of the Texas Revised Inventory of Grief, *IPGDS* sum value of the International Prolonged Grief Disorder Scale, *PSS* Sum value of the Perceived Stress Scale, *BRS* Mean of the Brief Resilience Scale, *WHO-5* Sum value of World Health Organization Well-Being-Index, *F-SozU* Sum Value of the Brief Perceived Social Support Questionnaire, *ASKU* Mean of the short form of the Self-Efficacy Scale.

### Frequency and correlations of loss- and restoration-oriented stressors

Table 1 shows the respective rates of participants who reported experiencing COVID-specific loss-oriented and restoration-oriented stressors. Particularly frequent loss-oriented stressors were not being able to say goodbye (n = 262, 53.5%) and being prohibited from physical contact with the deceased due to COVID restrictions (n = 247, 50.4%). The fact of not being able to attend the funeral of the deceased was mentioned least frequently (n = 24, 4.9%). The two most frequently mentioned restoration-oriented stressors were separation from one's social environment (n = 393, 80.2%) and longing for the “normalcy” of life before the pandemic (n = 390, 79.6%). Perceived stigma due to a loss to SARS-CoV-2 infection was mentioned least frequently (n = 17, 3.5%). Participants reported an average of 7.30 COVID-specific loss-oriented stressors out of 21 stressors queried (SD = 3.17, Min = 1, Max = 16) and 6.99 restoration-oriented stressors out of 19 stressors queried (SD = 2.54, Min = 0, Max = 15).

### Research question 1: cross-sectional predictors of acute grief intensity (TRIG)

In the first step of the regression, sociodemographic and loss-related variables explained 25% of the variance. In the second step, the inclusion of COVID-specific loss- and restoration-oriented stressors added 8% of explained variance and the inclusion of stress variables in the third step added another 15% of explained variance. The latter was the best-fitting model, as the inclusion of resilience factors in the fourth step did not further improve the model fit. Full model statistics are reported in Table 4.

**Table 4:** Regression Model for acute grief intensity (TRIG) T0.

Predictor	B	SE	β	p
Step 1: R^2^ = 0.26, Corrected R^2^ = 0.25, ΔR^2^ = 0.26, p < 0.001, F (df) = 24.79 (6, 423)				
Constant term	1.60	0.26		< 0.001
Age	0.01	0.00	0.16	< 0.001
Age deceased	0.02	0.00	0.37	< 0.001
Days since loss	0.00	0.00	0.08	0.066
Relationship quality	− 0.01	0.00	− 0.20	< 0.001
Death by natural cause	0.11	0.08	0.06	0.186
Death by SARS-CoV-2	− 0.33	0.08	− 0.18	< 0.001
Step 2: R^2^ = 0.34, Corrected R^2^ = 0.33, ΔR^2^ = 0.08, p < 0.001, F (df) = 26.86 (8, 421)				
Constant term	2.34	0.27		< 0.001
Age	0.01	0.00	0.11	0.008
Age deceased	0.02	0.00	0.39	< 0.001
Days since loss	0.00	0.00	0.11	0.010
Relationship quality	− 0.01	0.00	− 0.19	< 0.001
Death by natural cause	0.07	0.08	0.04	0.398
Death by SARS-CoV-2	− 0.06	0.09	− 0.03	0.531
Loss-oriented stressors	− 0.06	0.01	− 0.24	< 0.001
Restoration-oriented stressors	− 0.05	0.01	− 0.16	< 0.001
Step 3: R^2^ = 0.49, Corrected R^2^ = 0.48, ΔR^2^ = 0.15, p < 0.001, F (df) = 40.07 (10, 419)				
Constant term	2.59	0.30		< 0.001
Age	0.01	0.00	0.07	0.056
Age deceased	0.01	0.00	0.29	< 0.001
Days since loss	0.00	0.00	0.05	0.153
Relationship quality	− 0.01	0.00	− 0.20	< 0.001
Death by natural cause	0.11	0.07	0.06	0.122
Death by SARS-CoV-2	− 0.16	0.08	− 0.09	0.044
Loss-oriented stressors	− 0.04	0.01	− 0.16	< 0.001
Restoration-oriented stressors	− 0.01	0.01	− 0.02	0.647
WHO-5	0.04	0.01	0.28	< 0.001
PSS	− 0.02	0.01	− 0.20	< 0.001

TRIG: Mean of the TRIG questionnaire (range: 1–5), lower values indicate higher grief intensity. Age: continuous age variable (min 18, max 80 years), Age deceased: continuous age variable (min 0, max 100 years), Days since loss: Number of days between date of participation and date of loved one’s death, Relationship quality: VAS (0 = very poor, 100 = very good), Death by natural cause: dichotomized variable (0 = no, 1 = yes), Death by SARS-CoV-2: dichotomized variable (0 = no, 1 = yes), Loss-oriented stressors: sum value of agreed loss-oriented stressors, Restoration-oriented stressors: sum value of agreed restoration-oriented stressors, WHO-5 = (WHO-5 Well-Being Index) Summed value of the scale, with lower values corresponding to a lower sense of well-being, PSS = Perceived Stress Scale (10 items), with higher values indicating higher stress experience.

Higher exposure to loss-related stressors (but not restoration-oriented stressors) was cross-sectionally associated with more pronounced acute grief intensity at T0 (B = − 0.04, SE = 0.01, β = − 0.16, p < 0.001). Additionally, a more intense acute grief response correlated with younger age of the deceased, better relationship quality with the deceased, loss due to SARS-CoV-2 infection, poorer well-being, and higher perceived stress. In accordance with Cohen^34^, the final regression model for the prediction of acute grief (TRIG) at T0 shows a moderate explanation of variance, with R^2^ = 0.48.

### Research question 2: longitudinal predictors of prolonged grief intensity (IPGDS)

In the first step, sociodemographic variables explained 23% of the variance in prolonged grief at T1. The inclusion of acute grief intensity at T0 significantly improved the model fit by 43% explained variance. Adding the COVID-specific loss- and restoration-oriented stressors did not result in a significantly better model fit (2% explained variance). Adding the variables mental well-being and perceived stress improved the model by a further 7% explained variance. This latter model also resulted in the best fit, with a high explanation of variance, at R^2^ = 0.71. Comprehensive model statistics can be found in Table 5. The only significant longitudinal predictors of higher prolonged grief intensity at T1 were acute grief intensity at T0 (B = − 8.20, SE 1.18, β = − 0.55, p < 0.001) and poorer mental well-being at T0 (B = − 0.52, SE = 0.17, β = − 0.25, p = 0.004). Neither loss-oriented nor restoration-oriented stressors during the COVID-19 pandemic significantly predicted prolonged grief intensity in the final model. It is noteworthy that restoration-oriented stressors seemed to be more relevant than loss-oriented stressors in the third regression step, although this effect was resolved by the inclusion of additional variables. Adding resilience factors also did not result in a better model fit in the longitudinal model. Control analysis with the imputed data resulted in similar findings: Acute grief intensity at T0 was one of two significant predictors of higher prolonged grief intensity at T1, stressors in terms of the DPM during the COVID-19 pandemic showed no significant influence, and again protective factors did not further improve the model. However, rather than poorer mental well-being showing to be a significant predictor of higher prolonged grief, higher levels of stress emerged here as significant predictor (B = 0.25, SE = 0.08, β = 0.19, p = 0.011; see: Table 5 in the supplementary material).

**Table 5:** Regression model for prolonged grief intensity (IPGDS) T1.

Predictor	B	SE	β	p
Step 1: R^2^ = 0.23, Corrected R^2^ = 0.17, ΔR^2^ = 0.23, p = 0.002, F (df) = 3.68 (7, 85)				
Constant term	53.13	8.94		< 0.001
Age	− 0.16	0.10	− 0.16	0.103
Age deceased	− 0.24	0.07	− 0.38	0.001
Days since loss	− 0.00	0.01	− 0.02	0.818
Relationship quality	0.08	0.07	0.11	0.254
Death by natural cause	− 0.84	2.82	− 0.03	0.766
Death by SARS-CoV-2	4.94	2.89	0.17	0.091
Recent death	1.13	5.06	0.02	0.823
Step 2: R^2^ = 0.66, Corrected R^2^ = 0.63, ΔR^2^ = 0.43, p < 0.001, F (df) = 104.40 (1, 84)				
Constant term	72.81	6.31		< 0.001
Age	− 0.03	0.07	− 0.03	0.637
Age deceased	− 0.07	0.05	− 0.11	0.155
Days since loss	0.00	0.01	0.04	0.558
Relationship quality	− 0.04	0.05	− 0.06	0.358
Death by natural cause	0.20	1.90	0.01	0.917
Death by SARS-CoV-2	0.91	1.98	0.03	0.648
Recent death	3.76	3.41	0.07	0.273
TRIG	− 11.36	1.11	− 0.76	< 0.001
Step 3: R^2^ = 0.68, Corrected R^2^ = 0.64, ΔR^2^ = 0.02, p = 0.105, F (df) = 2.31 (2, 82)				
Constant term	65.49	7.17		< 0.001
Age	− 0.01	0.07	− 0.01	0.871
Age deceased	− 0.08	0.05	− 0.12	0.123
Days since loss	0.00	0.01	0.03	0.632
Relationship quality	− 0.04	0.05	− 0.06	0.384
Death by natural cause	0.43	1.87	0.02	0.819
Death by SARS-CoV-2	− 0.24	2.17	− 0.01	0.911
Recent death	3.75	3.36	0.07	0.267
TRIG	− 10.71	1.16	− 0.72	< 0.001
Loss-oriented stressors	0.11	0.27	0.03	0.690
Restoration-oriented stressors	0.63	0.31	0.14	0.044
Step 4: R^2^ = 0.74, Corrected R^2^ = 0.71, ΔR^2^ = 0.07, p < 0.001, F (df) = 10.43 (2, 80)				
Constant term	59.09	7.90		< 0.001
Age	− 0.01	0.06	− 0.01	0.841
Age deceased	− 0.07	0.04	− 0.11	0.123
Days since loss	0.00	0.01	0.05	0.365
Relationship quality	− 0.01	0.04	− 0.02	0.779
Death by natural cause	− 0.52	1.70	− 0.02	0.760
Death by SARS-CoV-2	1.25	1.98	0.04	0.529
TRIG	− 8.20	1.18	− 0.55	< 0.001
Recent death	4.49	3.04	0.09	0.144
Loss-oriented stressors	0.05	0.25	0.01	0.834
Restoration-oriented stressors	0.32	0.29	0.07	0.280
WHO-5	− 0.52	0.17	− 0.25	0.004
PSS	0.17	0.13	0.11	0.201

IPGDS: sum value of the International Prolonged Grief Disorder Scale, higher values indicate higher grief intensity. Age: continuous age variable (min 18, max 80 years), Age deceased: continuous age variable (min 0, max 100 years), Days since loss: Number of days between date of participation and date of loved one’s death, Relationship quality: VAS (0 = very poor, 100 = very good), Death by natural cause: dichotomized variable (0 = no, 1 = yes), Death by SARS-CoV-2: dichotomized variable (0 = no, 1 = yes), Recent death: dichotomized variable (0 = no, 1 = yes), TRIG: Mean of the TRIG questionnaire (range: 1–5), lower values indicate higher grief intensity, Loss-oriented stressors: sum value of agreed loss-oriented stressors, Restoration-oriented stressors: sum value of agreed restoration-oriented stressors, WHO-5 = (WHO-5 Well-Being Index) Summed value of the scale, with lower values corresponding to a lower sense of well-being, PSS = Perceived Stress Scale (10 items), with higher values indicating higher stress experience.

## Discussion

The present study is one of the first to examine the longitudinal influence of COVID-specific loss- and restoration-oriented stressors^7^ on the grieving process. These stressors were widespread in our sample. Besides loss-oriented stressors, further significant cross-sectional associations with acute grief emerged with younger age of the deceased, better relationship quality with the deceased, loss due to SARS-CoV-2 infection, poorer mental well-being, and higher perceived stress. Longitudinally, only acute grief intensity at T0 and mental well-being at T0 predicted higher prolonged grief intensity at T1 6 months later. The assumed protective factors (resilience, perceived social support, and self-efficacy) did not significantly correlate with grief at any measurement time point.

The frequencies of the individual stressors varied considerably. For instance, restoration-oriented stressors such as separation from friends (80.2%), longing for "normalcy" (79.6%), and restrictions of leisure time (73.3%) were particularly common. An online survey by Selman et al.^13^ found comparable frequencies in a British sample, with 80.7% of participants reporting limited contact with close family members and friends. With regard to loss-oriented stressors, however, there were notable differences: A UK study reported a higher degree of stress due to restrictions in funeral arrangements (93.4% vs. 62.7% in our sample), the lack of ability to say goodbye (63.9% vs. 53.5% in our sample) and a lack of opportunity to visit the deceased prior to death (54.3% vs. 34.3% in our sample). These discrepancies might be explained by the varying measures to curb the pandemic in the different countries. It can be stated that the assumed COVID-specific stressors were frequently experienced by bereaved individuals in general—and not only those whose significant other had died of or with SARS-CoV-2.

Acute and prolonged grief intensity were predicted by different variables. The cross-sectional analysis revealed a wide range of factors associated with acute grief, such as good relationship quality with the deceased in line with other COVID-specific grief studies^13,14,35,36^. Interestingly, only the COVID-specific loss-oriented stressors (and not the restoration-oriented stressors) significantly correlated with acute grief. This aligns with the findings of Neimeyer and Lee^37^, suggesting that the circumstances of the loss experience may explain a high degree of pandemic grief. The DPM assumes that mourners switch between loss- and restoration-oriented processes, but the time courses and interactions between the two orientations have not yet been well studied. Nevertheless, it stands to reason that mourners focus more on loss-related burdens shortly after the loss. A recent study by Eisma et al.^38^ examined the prevalence and correlates of restoration-oriented stressors using the newly developed Restoration-Oriented Stressors Inventory (ROSI). The results revealed no direct correlation between restoration-oriented stressors and prolonged grief in the final model. However, the stressors did lead to increased stress in the participants. It is possible that restoration-oriented stressors are not directly associated with grief per se, but rather render coping with grief more difficult by increasing stress during the grieving process. At the same time, there may be a COVID-specific mechanism underlying this finding: In a qualitative study by Buckley et al.^12^, the authors suggested that bereaved individuals had less daily routine and social support during the pandemic, which may have disrupted the oscillation process, leaving mourners "stuck" in their grief. Thus, the results of our study may also indicate that affected individuals had fewer opportunities to return to their old lives, potentially making restoration-oriented stressors less relevant during this time. According to our data, one may tentatively suggest that restoration-oriented stressors gain relevance from a longitudinal perspective and with regard to prolonged grief. Concerning these different explanatory approaches, it would be interesting for future research to study the temporal progression of the relevance of the loss- and restoration-oriented stressors. In the longitudinal analysis, only acute grief and mental well-being at baseline emerged as predictors of prolonged grief in the final model. The acute grief reaction is already known to be a particularly relevant predictor of later grief intensity^33^. While COVID-specific stressors did not significantly affect prolonged grief intensity, since loss-oriented stressors were significantly associated with the acute grief reaction, there may be an indirect relationship: Higher loss-oriented stress appears to be associated with increased acute grief intensity, which in turn seems to be a relevant predictor of prolonged grief intensity. Thus, the long-term effect of loss-oriented stressors may possibly be mediated by the short-term effect of acute grief.

Our analyses revealed no significant association between the hypothesized protective factors and grief. In contrast, in a sample of individuals who had lost a significant other during the pandemic, Skalski et al.^39^ found that resilience mediated the relationship between social support and dysfunctional grief. A systematic review by Scott et al.^40^ found an association between social support and depression after a loss, whereas the evidence for an association between social support and prolonged grief responses was conflicting. It is possible that the effect of social support was suppressed during the COVID-19 pandemic because the measures to curb the pandemic required a withdrawal from direct social contacts, and social support could not be experienced in the usual way, e.g., through physical contact and proximity. Compared to the norm scores and validation samples, our sample has overall rather low scores on all three scales^24,26,31^. According to the negativity bias, negative experiences have a more pronounced effect on psychological states than do positive experiences—an effect that has been demonstrated in many different contexts^41^. The present findings may therefore suggest that important resources in the grieving process were eliminated during the COVID-19 pandemic, with psychological stress exerting an even greater impact on affected individuals. Yet, it is also possible that no significant correlations could be found due to the choice of questionnaires. For example, the German Brief Perceived Social Support Questionnaire (F-SozU) items might not reflect well the constrictions during the COVID-19 measures. And the query of resilience as stable trait might also be considered problematic with regard to the dynamic process of coping with grief. Moreover, self-efficacy is queried here as a one-time measurement, although self-efficacy can vary greatly depending on the context. A third explanation centers on the paradox described by Bonanno^15^: It is possible that coping with grief is rather a flexible process of adaptation, therefore single resources could not represent a resilient grief process well at all. This would also fit into the concept of DPM, which assumes an oscillation process in coping with grief—suggesting a process in which mourners sometimes turn more and sometimes less to the stressors in order to cope with them.

To minimize distress due to the participation in our study, we followed the recommendations for online grief studies proposed by Smith et al.^16^. One “safety measure” was a check-in email, which we sent to all participants a few days after their participation. Only a small number of participants (n = 15) replied to the email, thanking us for our interest in this topic and the opportunity to share their experiences. Of these, seven participants expressed a feeling of being forgotten by politicians and society, or that other topics were seen as more relevant—a concept known as “disenfranchised grief”^9^. Similarly, Albuquerque et al.^42^ suggested that during the pandemic, there was insufficient space for bereaved individuals to grieve in their community. The responses to our email may indicate that there is still a need for more public recognition of these losses and that many bereaved persons feel alone in their grief.

Some limitations that restrict the generalizability of our findings should be mentioned. Most of our sample consisted of females, thus reducing the generalizability of gender-specific effects. It is also difficult to assess the extent to which our survey is representative of the totality of bereaved individuals. In addition, a relevant self-selection bias may be present, insofar as people who cared about sharing their personal experience were more likely to participate. Moreover, as a comprehensive and validated list of loss- and restoration-oriented stressors did not yet exist when we began the study, we classified the stressors according to the DPM, creating total scores to evaluate the respective stressor burdens. A recent study by Eisma et al.^38^ examined the newly developed Restoration-Oriented Stressors Inventory. While our questions on restoration-oriented stressors show some similarities with this Inventory, there are also some significant differences. It would therefore be interesting to further validate restoration-oriented stressors. It is possible that the COVID-specific stress factors show a significantly different factor structure, which deviates from the classification into restoration- and loss-oriented stressors, and that the stressors exhibit unknown interactions among each other.

According to our findings, COVID-specific stressors affected grief responses in the assessed sample. A direct main effect on prolonged grief intensity was not found, although the long-term effect of loss-related stressors may be mediated by the effect on acute grief. In particular, loss-oriented stressors played a role in coping with acute grief cross-sectionally. Previous studies with counselors and psychotherapists of bereaved individuals during the COVID-19 pandemic indicate that these COVID-specific issues also occupied a large space in the support provided to bereaved individuals during the pandemic^11,12^, with the loss of resources and support standing out as an aggravating factor in coping with grief. As a practical implication, therefore, it can be deduced that the COVID-19 pandemic was relevant to mourners and that these circumstances should thus also be taken into account in terms of support provision. In this regard, interventions oriented to the DPM appear to be effective in supporting bereaved individuals. Further studies should further investigate the influence of pandemic stressors on the course of grief—especially with regard to whether a classification into loss- and restoration-oriented stressors is meaningful in such contexts. Moreover, studies on how grievers can be supported at this particular time, encompassing these extraordinary stressors, would also be fruitful. On a societal level, it is important to publicly acknowledge the losses of bereaved individuals and to create spaces in which they can share their experiences.

### Supplementary Information

Supplementary Table S1.

## Data Availability

The datasets generated and analysed during the current study are available from the corresponding author on reasonable request.
