# Supplementary material for: Influence of loss- and restoration-oriented stressors on grief in times of COVID-19
Source: Sci Rep. 2023 Nov 9;13:19584. doi: 10.1038/s41598-023-46403-6 (PMC10638438; doi:10.1038/s41598-023-46403-6)
Supplement: Supplementary file 1 — Supplementary Table S1. [file 41598_2023_46403_MOESM1_ESM.pdf]

Influence of loss- and restoration-oriented stressors on grief in times of COVID-19
Supplementary table by S. Palm, B. K. Doering, T. Kubiak, K. Geschke, A. Fellgiebel, A. Wuttke

Supplementary table S1: Regression model for prolonged grief intensity (IPGDS) of the imputed data at T1.

**Predictor**	**B**	**SE**	**β**	**p**
**Step 1:** $R^2 = 0.16$, Corrected $R^2 = 0.14$, $\Delta R^2 = 0.16$, $p < 0.001$, F (df) = 8.67 (7,329)				
Constant term	52.24	3.69		< 0.001
Age	-0.11	0.04	-0.14	0.008
Age deceased	-0.14	0.03	-0.29	< 0.001
Days since loss	-0.01	0.00	-0.13	0.012
Relationship quality	0.02	0.03	0.04	0.438
Death by natural cause	-1.85	1.24	-0.09	0.137
Death by SARS-CoV-2	3.19	1.16	0.15	0.006
Recent death	0.15	1.83	0.00	0.937
**Step 2:** $R^2 = 0.47$, Corrected $R^2 = 0.46$, $\Delta R^2 = 0.31$, $p < 0.001$, F (df) = 36.20 (8,328)				
Constant term	62.15	3.02		< 0.001
Age	-0.02	0.03	-0.02	0.646
Age deceased	-0.02	0.02	-0.04	0.374
Days since loss	-0.01	0.00	-0.08	0.065
Relationship quality	-0.03	0.02	-0.06	0.173
Death by natural cause	-1.23	0.98	-0.06	0.211
Death by SARS-CoV-2	1.36	0.93	0.06	0.146
Recent death	1.41	1.46	0.04	0.335
TRIG	-7.82	0.56	-0.65	< 0.001
**Step 3:** $R^2 = 0.47$, Corrected $R^2 = 0.46$, $\Delta R^2 = 0.01$, $p = 0.203$, F (df) = 29.39 (10,326)				
Constant term	59.56	3.51		< 0.001
Age	-0.01	0.03	-0.01	0.816
Age deceased	-0.02	0.03	-0.05	0.364
Days since loss	-0.01	0.00	-0.08	0.061
Relationship quality	-0.03	0.02	-0.06	0.169
Death by natural cause	-1.02	0.99	-0.05	0.302
Death by SARS-CoV-2	1.14	1.06	0.05	0.286
Recent death	1.34	1.46	0.04	0.356
TRIG	-7.61	0.60	-0.63	< 0.001
Loss-oriented stressors	-0.03	0.14	-0.01	0.849
Restoration-oriented stressors	0.29	0.16	0.08	0.075
**Step 4:** $R^2 = 0.51$, Corrected $R^2 = 0.49$, $\Delta R^2 = 0.04$, $p < 0.001$, F (df) = 28.05 (12,324)				
Constant term	51-04	4.24		< 0.001
Age	0.01	0.03	0.01	0.831
Age deceased	-0.02	0.02	-0.04	0.416
Days since loss	-0.00	0.00	-0.07	0.108
Relationship quality	-0.01	0.02	-0.03	0.519
Death by natural cause	-1.24	0.96	-0.06	0.200
Death by SARS-CoV-2	1.71	1.04	0.08	0.101
Recent death	1.45	1.42	0.04	0.308
TRIG	-6.18	0.65	-0.51	< 0.001
Loss-oriented stressors	-0.08	0.14	-0.03	0.565
Restoration-oriented stressors	0.02	0.16	0.01	0.903
WHO-5	-0.13	0.09	-0.08	0.157
PSS	0.25	0.08	0.19	0.001
**Step 5:** R2 = 0.52, Corrected R2 = 0.49, Δ R2 = 0.01, $p = 0.344$, F (df) = 22.690 (15,321)				
Constant term	54.56	4.95		< 0.001
Age	0.01	0.03	0.01	0.830
Age deceased	-0.02	0.02	-0.04	0.455
Days since loss	-0.01	0.00	-0.08	0.069
Relationship quality	-0.01	0.02	-0.01	0.760
Death by natural cause	-1.24	0.97	-0.06	0.201
Death by SARS-CoV-2	1.82	1.05	0.09	0.083
Recent death	1.51	1.42	0.04	0.288
TRIG	-6.07	0.66	-0.50	< 0.001
Loss-oriented stressors	-0.08	0.14	-0.03	0.542
**Predictor**	**B**	**SE**	**β**	**p**

Restoration-oriented stressors	-0.01	0.16	-0.00	0.949
WHO-5	-0.10	0.09	-0.06	0.264
PSS	0.20	0.08	0.16	0.011
BRS	-0.89	0.57	-0.09	0.121
ASKU	0.07	0.62	0.01	0.908
Social Support	-0.05	0.07	0.03	0.517

Annotations: IPGDS: sum value of the International Prolonged Grief Disorder Scale, higher values indicate higher prolonged grief intensity, missing values were substituted by the pooled mean of the estimates obtained via multiple imputation. Age: continuous age variable (min 18, max 80 years), Age deceased: continuous age variable (min 0, max 100 years), Days since loss: Number of days between date of participation and date of loved one's death, Relationship quality: VAS (0 = very poor, 100 = very good), Death by natural cause: dichotomized variable (0 = no, 1 = yes), Death by SARS-CoV-2: dichotomized variable (0 = no, 1 = yes), Recent death: dichotomized variable (0 = no, 1 = yes) missing values were substituted by the pooled mode of the estimates obtained via multiple imputation, TRIG: Mean of the TRIG questionnaire (range: 1-5), lower values indicate higher acute grief intensity, Loss-oriented stressors: sum value of agreed loss-oriented stressors, Restoration-oriented stressors: sum value of agreed restoration-oriented stressors, WHO-5 = (WHO-5 Well-Being Index) Summed value of the scale, with lower values corresponding to a lower sense of well-being, PSS = Perceived Stress Scale (10 items), with higher values indicating higher stress experience, BRS = mean of the Brief Resilience Scale, with higher values indicating higher resilience, ASKU = sum score of the General Self-Efficacy Short Scale, with higher values indicating higher self-efficacy, Social Support = sum score of the German Brief Perceived Social Support Questionnaire, with higher values indicating higher perceived social support.
